# A Study of 11-[^3^H]-Tetrodotoxin Absorption, Distribution, Metabolism and Excretion (ADME) in Adult Sprague-Dawley Rats

**DOI:** 10.3390/md15060159

**Published:** 2017-06-02

**Authors:** Bihong Hong, Hui Chen, Jiacai Han, Quanling Xie, Jianlin He, Kaikai Bai, Yanming Dong, Ruizao Yi

**Affiliations:** 1 Department of Materials Science and Engineering, College of Materials, Xiamen University, Xiamen 361005, China; bhhong@tio.org.cn; 2 Engineering Research Center of Marine Biological Resource Comprehensive Utilization, Third Institute of Oceanography, State Oceanic Administration, Xiamen 361005, China; chenhui@tio.org.cn (H.C.); qlxie@tio.org.cn (Q.X.); jlhe@tio.org.cn (J.H.); kkbai@tio.org.cn (K.B.); 3 Department of Inspection and Quarantine of Goods, Pingtan Entry-Exit Inspection & Quarantine Bureau of P.R.C, Pingtan 350400, China; ytt629n@163.com

**Keywords:** tetrodotoxin, ADME, TTX, 11-[^3^H]TTX, non-clinical pharmacokinetics

## Abstract

Tetrodotoxin (TTX) is a powerful sodium channel blocker that in low doses can safely relieve severe pain. Studying the absorption, distribution, metabolism and excretion (ADME) of TTX is challenging given the extremely low lethal dose. We conducted radiolabeled ADME studies in Sprague-Dawley rats. After a single dose of 6 μg/(16 μCi/kg) 11-[^3^H]TTX, pharmacokinetics of plasma total radioactivity were similar in male and female rats. Maximum radioactivity (5.56 ng Eq./mL) was reached in 10 min. [^3^H]TTX was below detection in plasma after 24 h. The area under the curve from 0 to 8 h was 5.89 h·ng Eq./mL; mean residence time was 1.62 h and t_½_ was 2.31 h. Bile secretion accounted for 0.43% and approximately 51% of the dose was recovered in the urine, the predominant route of elimination. Approximately 69% was recovered, suggesting that hydrogen tritium exchange in rats produced tritiated water excreted in breath and saliva. Average total radioactivity in the stomach, lungs, kidney and intestines was higher than plasma concentrations. Metabolite analysis of plasma, urine and feces samples demonstrated oxidized TTX, the only identified metabolite. In conclusion, TTX was rapidly absorbed and excreted in rats, a standard preclinical model used to guide the design of clinical trials.

## 1. Introduction

Tetrodotoxin (TTX) is a potent neurotoxin found in a variety of marine and terrestrial species [1,2,3,4,5,6,7,8]. Clinical studies suggest that low doses of TTX can safely relieve severe cancer-related pain [9,10,11] and reduce cue-induced drug craving and anxiety in abstinent heroin addicts [12]. TTX is a powerful sodium channel blocker [13] and the median lethal dose (LD_50_) is about 10 μg/kg for adult Sprague-Dawley (SD) rats with intramuscular injection. After administration, the amount of TTX in the blood, urine and tissues is extremely low, as well as its metabolites. Both are very polar compounds, which may cause the problem of ion suppression in mass spectrometry (MS) analysis. Therefore, a very sensitive method is required. In recent years, liquid chromatography-mass spectrometry (LC-MS) and especially LC-MS/MS are regarded as the most popular and sensitive methods for determination of TTX in plasma and urine [14]. Jen et al. [15] used solid-phase extraction combined with LC-MS/MS to analyze trace amounts of TTX in patients’ serum. The observed limit of detection (LOD) and the limit of quantification (LOQ) were 0.1 and 1 ng/mL in serum, respectively. Tsai et al. [16] developed a new protocol to determine TTX in the urine and blood of human subjects. The samples were cleansed using a C18 Sep-Pak cartridge column and determined by LC-MS. The LOD was 15.6 nM (5.0 ng/mL). Fong et al. [17] developed a method that combined double solid phase extraction (C18 and hydrophilic interaction liquid chromatography) and analyzed by LC-MS/MS. The LOD and LOQ were 0.13 and 2.5 ng/mL, respectively. Saito et al. [18] developed a validated method to determine the concentration of TTX by using MonoSpin CBA or amide column, with the help of LC-MS/MS analysis. The LOQ for the TTX in serum and urine were 1 and 0.5 ng/mL, respectively. Tsujimura et al. [19] separated and identified TTX of human serum by the combination of liquid chromatography (LC) equipped with a multi-mode ODS column and tandem mass spectrometry. The LOQ was extremely low as 0.5 ng/mL in serum. Studying the absorption, distribution, metabolism and excretion (ADME) of TTX in adult SD rats is much more challenging based on the best reported LODs for TTX to date.

Using radiolabeled drugs to study ADME has the advantages of high sensitivity because of low biological background. Watabe et al. [20] used the low specific radioactivity of [^3^H]TTX to investigate the anatomical locations of TTX in pufferfish. After intraperitoneal injection into the pufferfish, [^3^H]TTX accumulated in most tissues; the highest levels were observed in the skin followed by the liver, intestines and muscle. With time, the radioactivity levels decreased remarkably in most tissues except for the skin and gallbladder. Radioactivity measured in tissues in this study did not necessarily reflect the true TTX levels because TTX may be metabolized in the pufferfish. Liu et al. [21] used an isotopic tracer method to study the metabolic dynamics and distribution of TTX in mice after administration of [^125^I]TTX intravenously. The content of [^125^I]TTX reached the highest concentration in the blood and lungs after 0.17 h. With time, the content increased in the liver and reached its highest concentration at 24 h. TTX metabolites were present mainly in the liver. These methods provide a reference for the study of pharmacokinetics of TTX, but it does not meet the requirements of the guidelines for the study of non-clinical pharmacokinetics of drugs. We wanted to explore the ADME of TTX using a standard model, the Sprague-Dawley rat. The objectives of the present study were to determine the pharmacokinetics (PK) of TTX in SD rats; to identify and quantify metabolites in plasma and excreta; and to determine the rates and routes of excretion of [^3^H]TTX-related radioactivity, including mass balance in excreta.

## 2. Results

### 2.1. Observation of Experimental Animal Health Status

No paradoxical reactions were observed during the experiments after a single intramuscular dose of 6 μg/(16 μCi/kg) 11-[^3^H]TTX in male or female rats. Thus, this dose of [^3^H]TTX was well tolerated in SD rats.

### 2.2. Stability of [^3^H]TTX

The stability of 11-[^3^H]TTX was studied before and after administration. As shown in Table 1, the radiochemical purity of 11-[^3^H]TTX remained about 90% both pre-dose and post-dose, with a average purity of 90.48%, while it took only four hours on the TTX administration. It suggested the stability of 11-[^3^H]TTX was guaranteed during TTX administration.

### 2.3. Pharmacokinetics of Radioactivity and TTX

**Figure 1:**
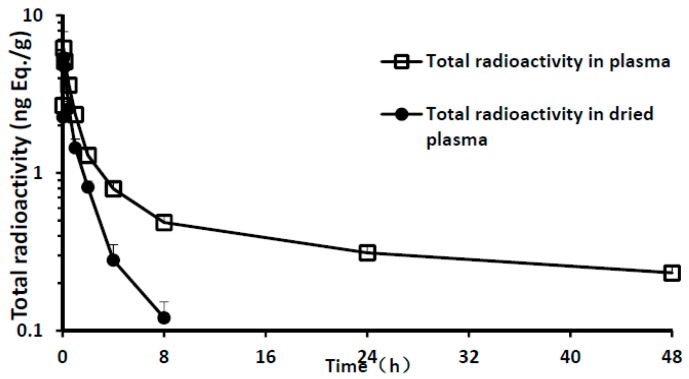
Total radioactivity and time curve of plasma samples (mean of three male and three female rats).

Given the hydrogen-tritium exchange of 11-[^3^H]TTX in the plasma and that formed tritiated water would interfere with the results of analysis, plasma samples were dried to remove tritiated water. The plasma drying loss rate reached 65% after 4 h and 100% after 24 h (Table 2). Total radioactivity in plasma was analyzed before and after drying. The results showed that the total radioactivity of tritiated water in plasma increased gradually with the prolonging of time after administration (Figure 1). Pharmacokinetics (PK) parameters using the total radioactivity in dried plasma were calculated. A summary of the PK parameters of total radioactivity in the plasma is presented in Table 3. After a single intramuscular dose of 6 μg/(16 μCi/kg) 11-[^3^H]TTX, the PK characteristics of plasma total radioactivity in male and female rats were similar. The maximum radioactivity C_max_ was reached after 10 min; mean C_max_ was 5.56 ng Eq./mL. The elimination rapidly peaked and radioactivity in the plasma was unable to be detected 24 h after administration. The area under the curve from 0 to 8 h (AUC_0–8 h_) was 5.89 h·ng Eq./mL, The mean residence time (MRT) was 1.62 h and t_½_ was 2.31 h.

### 2.4. Excretion of [^3^H]TTX in Urine and Feces

After a single intramuscular dose of 6 μg/(16 μCi/kg) 11-[^3^H]TTX in male and female rats, the cumulative recovery rates of radioactivity in urine, feces, cage wash and carcass are summarized in Table 4. The mean recovery rate of total radioactivity was 69.35% between 0 and 72 h, and 51.16% was recovered in the urine, 3.87% in the feces, 10.01% in the cage wash, and 4.31% in the carcass. After 72 h, the radioactivity of all samples was lower than the limit of quantification.

Meanwhile, the urine radioactivity before and after drying was calculated. Drying loss rates in urine are summarized in Table 5. Urine samples all contained a large amount of tritiated water, which increased gradually with the prolonging of time after administration.

### 2.5. Excretion of [^3^H]TTX in Bile

After a single intramuscular dose of 6 μg/(16 μCi/kg) 11-[^3^H]TTX in male and female with biliary duct cannulation (BDC) rats, cumulative recovery rates of radioactivity in bile, urine, feces and cage wash are summarized in Table 6. The mean recovery rate of total radioactivity was 65.58% between 0 and 72 h, except for the carcass; 0.43% was recovered in the bile, 57.26% in the urine, 0.94% in the feces, 6.94% in the cage wash, and 4.31% in the carcass.

### 2.6. Total Radioactivity in Tissues

**Figure 2:**
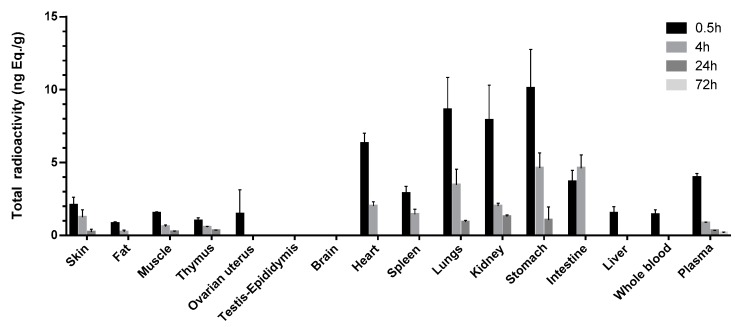
Distribution of [^3^H]TTX among blood, plasma and tissues in rats following intramuscular administration of [^3^H]TTX at different time points.

After a single intramuscular dose of 6 μg/(16 μCi/kg) 11-[^3^H]TTX in male and female rats, the mean total radioactivity concentrations distributed in whole blood, plasma and tissue-time are shown in Figure 2. The distribution concentrations of testis epididymis and whole brain at time points of 0.5, 4, 24 and 72 h were lower than the limit of quantification. The highest concentration of the total radioactivity in the other tissues was the highest at the first sampling point (0.5 h), except T_max_ for the intestinal wall, which was at 4 h. Total radioactivity was mainly distributed in the gastrointestinal tract wall after intramuscular administration at 0.5 h. The average total radioactivity in the stomach wall, lung, kidney, heart and intestinal wall were higher than in or near the point of time in plasma concentrations (3.97 ng Eq./g), 2.54, 2.17, 1.99, 1.59 and 0.924 times the plasma average total radioactivity, respectively. The total radioactivity in the other tissues was lower than the plasma concentrations, and the ovary, spleen, skin, skeletal muscle, liver, thymus and fat were from highest to lowest in the order of concentration. After 4 h, the total radioactivity of the intestinal wall reached C_max_, while C_max_ of the other tissues decreased. The average total radioactivity of the gastric intestinal wall, lung, kidney, heart, spleen and skin was higher than that time point of the plasma concentration (0.862 ng Eq./g), 5.37, 5.36, 4.03, 2.33, 2.33, 1.68 and 1.45 times of the plasma average total radioactivity, respectively. The total radioactivity of skeletal muscle, thymus and fat was lower than that of plasma, and the distribution of the liver and ovary uterus was lower than the lower limit of quantification. After 24 h, the tissue total radioactivity was significantly decreased. Only a small amount of radioactivity was detected in the kidney, gastric, lung, thymus, skeletal muscle and skin. At the last acquisition time point (72 h), the radioactivity of all detected tissues was lower than that of the lower limit of quantification.

### 2.7. Metabolite Identification and Profiling

**Figure 3:**
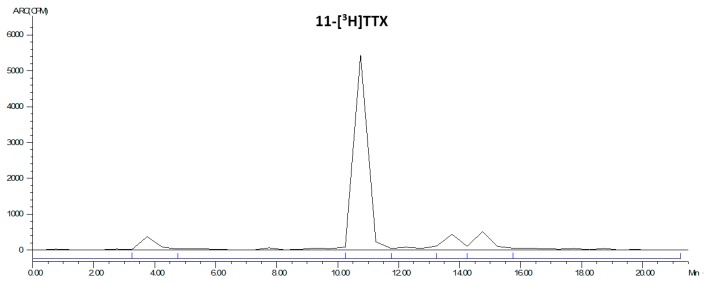
LC radiochromatogram of 11-[^3^H]TTX standard.

The LC radiochromatogram of the 11-[^3^H]TTX standard is shown in Figure 3.

#### 2.7.1. Plasma

**Figure 4:**
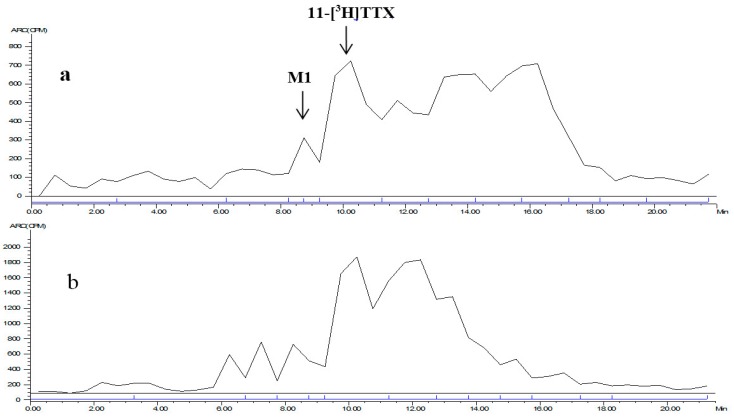
LC radiochromatogram of mixed plasma at: 0.083 h (**a**); and 4 h (**b**).

The mixed plasma radioactive metabolite spectrum of the male and female rats at time points of 0.083 and 4 h is shown in Figure 4. The radioactive metabolite ratio in total radioactivity (%HPLC) is shown in Table 7. After a single intramuscular dose of about 6 μg/(16 μCi/kg) 11-[^3^H]TTX, prototype drug (TTX) was detected in the mixed plasma accounting for total plasma exposure at time points of 0.083 h (female, 17.9%; male, 18.8%), 0.5 h (female, 25.9%; male 22.3%), 1 h (female, 18.2%; male, 21.0%), 2 h (female, 24.1%; male, 17.9%) and 4 h (female, 24.8%; male, 32.1%).

#### 2.7.2. Urine and Feces

**Figure 5:**
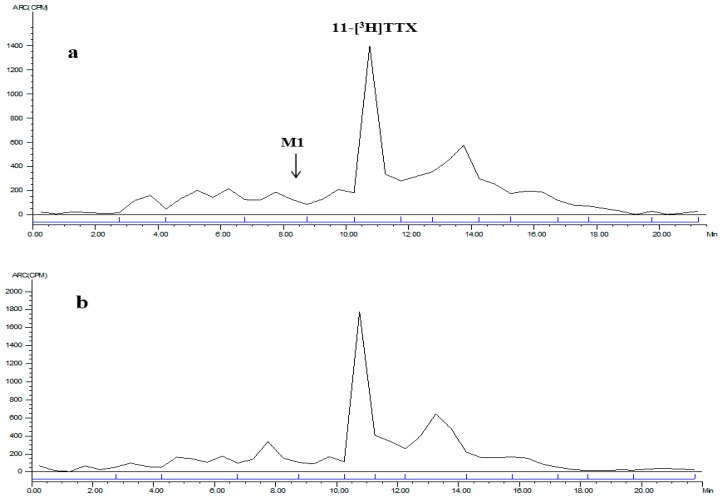
LC radiochromatogram of 0–8 h mixed urine for: female (**a**); and male (**b**) rats.

**Figure 6:**
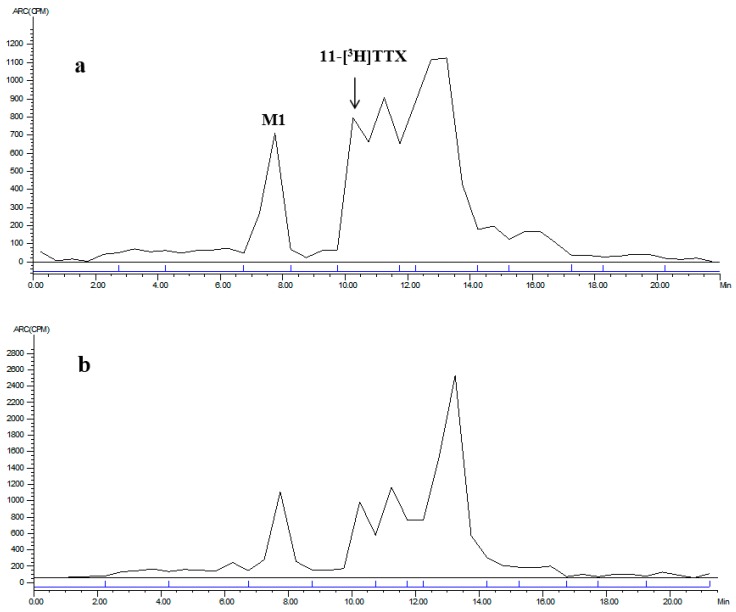
LC radiochromatogram of 0–24 h mixed feces for: female (**a**); and male (**b**) rats.

Mixed urine/feces radioactive metabolite spectra of the male and female rats between 0–8 h/0–24 h are shown in Figure 5 and Figure 6. The radioactive metabolites ratio in total radioactivity (%HPLC) is shown in Table 8. After a single intramuscular dose of about 6 μg/(16 μCi/kg) 11-[^3^H]TTX, the total radioactivity from the 0–8 h urine was 40.74% (female) and 52.64% (male), the prototype drug (TTX) accounted for 26.35% (female) and 26.11% (male), and M1 (monooxidized TTX) accounted for 7.15% (female) and 9.43% (male). Other metabolites were not identified, thus demonstrating that urine excretion is the predominant route of excretion of TTX. The total radioactivity from the 0–24 h feces was 2.65% (female) and 2.97% (male), the prototype drug (TTX) accounted for 28.19% (female) and 12.38% (male), and M1 (a single oxidation product of TTX) accounted for 10.73% (female) and 12.16% (male). Other metabolites were not identified.

#### 2.7.3. Identified Metabolites by LC-MS/MS

**Figure 7:**
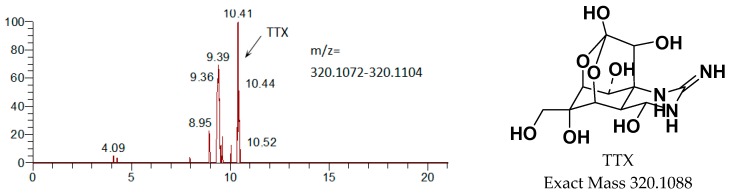

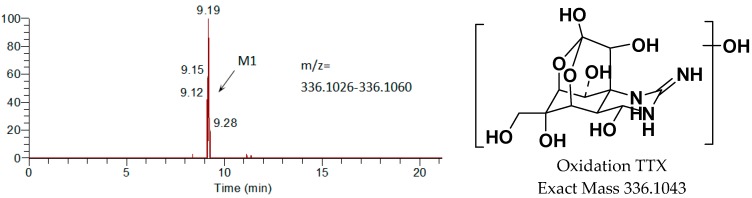
Mass-spectrometric identification of TTX metabolites in urine following intramuscular administration of [^3^H]TTX.

Metabolites were identified from urine and feces of male and female rats by LC-MS/MS. Except for the prototype drug (TTX), only one metabolite was identified, M1 (a single oxidation product of TTX). The main metabolites are shown in Figure 7. The radioactive peak at about 10.4 min was identified as TTX due to the following results of MS/MS: [M + H]^+^ *m/z* 320.1098 (calcd. 320.1088), [M + H − H_2_O]^+^ *m/z* 302 and [M + H − 2H_2_O]^+^ *m/z* 284. M1 as the metabolite of TTX, appeared at around 9.2 min, was oxidized TTX identified by analyzing the MS/MS results: [M1 + H]^+^ *m/z* 336.1054 (calcd. 336.1043), [M1 + H − H_2_O]^+^ *m/z* 318 and [M1 + H − 2H_2_O-O]^+^ *m/z* 286.

## 3. Discussion

TTX is a powerful neurotoxin attracting worldwide attention due to its strong sodium channel blocker activities. However, the reports focusing on ADME properties of TTX were rather poor. Only pufferfish [20,22,23,24] and mice [21] have been used as the model animals to systematically study the ADME of TTX. What is worse is the two models were improper for forecasing the ADME of TTX in human body.

The rat ADME model is widely used to predict metabolic products of drugs in humans. In this study, rat was utilized as the model animal for the first time to study the ADME of TTX. After intramuscular administration of 11-[^3^H]TTX, the [^3^H] signal could be found in most tissues, and its level remained high in some tissues such as intestine, stomach and lungs. This was in accordance with the previous report by Watabe [20]. The maximum radioactivity C_max_ in plasma was reached at 10 min after the administration of TTX. After that, the radio signal became weak rapidly and it was unable to be detected at 24 h after administration (Figure 2). The profile of TTX in the plasma of rats was consistent with that studies reported by Liu [21]. Besides, both TTX and the oxidized TTX as the metabolite of TTX, were found in the plasma, urine and feces of rats. Moreover, the majority of TTX was detected in the urine while only 0.43% of the radioactive dose was excreted in bile, suggesting urine excretion is the predominant route of elimination. It was clear TTX was excreted as the prototype drug.

Some interesting results of the study need deeper discussion. First, the distribution of TTX among different tissues was different between rats and other animals. During the study performed on the pufferfish named *Fugu rubripes*, the highest levels of TTX were observed in the intestine, followed by the skin, kidney and spleen. The levels decreased remarkably in most tissues except for the skin and gallbladder [20]. However, more TTX was observed in liver instead of skin on the pufferfish called *Takifugu rubripes* [22]. Furthermore, the preference of TTX distribution in some tissues to that in plasma was observed in rats. A surprisingly higher concentration of TTX was distributed in the stomach, lungs, kidney and heart than in the plasma at the point of 0.5 h. This may be partially explained by the abundant blood or a large amount of highly viscous material distributed in those tissues. It is also consistent with other investigators who have found that the highest concentration of intravenous administered [^125^I]TTX was distributed in the lung at the point of 0.5 h [21]. Finally, TTX was apt to be retained in intestines of animals. It had been reported TTX might be cumulative in the intestine of pufferfish after an intraperitoneal injection administration [20]. Similar results were observed in this experiment. The total radioactivity of [^3^H]TTX in the intestine of rats reached its peak at 4 h after the treatment while the [^3^H] signals of other tissues decreased. It may be related with the amount of receptor, but it remains uncertain why TTX accumulated in the intestines. Based on the analysis above, it suggested the ADME property of TTX among animal species may be different and complex. More investigation is needed.

The determination of TTX and its metabolites is one of technical barriers to be overcome. Although the highest dose of TTX (6 μg/kg) that could be tolerated by rats was given, the dose is extremely low in terms of detection. Meanwhile, TTX has extensive metabolism in animals, such as 4,9-anhydroTTX, 5,6,11-trideoxyTTX, 11-deoxyTTX and other oxidized TTXs [25,26], likely resulting in the awful low concentration of TTX metabolites in rats’ plasma, urine and feces. Therefore, most of the metabolites in the matrix could not be identified by LC-MS/MS. In addition to the LC-MS/MS, mass defect filtration (MDF) technique was actually applied by the authors to search the metabolic products of TTX in phase I and II in the urine samples and the results showed no mass peak of TTX related metabolites. Additionally, the identification of TTX metabolites is another challenge. Whether M1 was 11-oxoTTX was uncertain though these two compounds presented the same exact mass and the similar expected fragmentation law of MS. M1 might be 11-oxoTTX, which is a compound isolated from pufferfish, because the compound is a natural analog of TTX [27]. Exploring more suitable methods to identify TTX metabolites is challenging and it will be the subject of ongoing studies.

## 4. Materials and Methods

### 4.1. Regents

**Figure 8:**
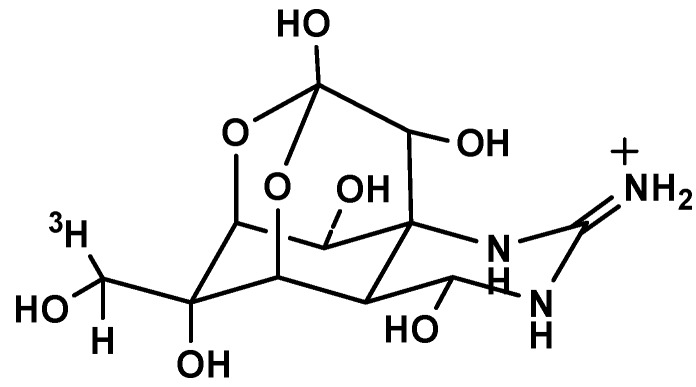
The structure of 11-[^3^H]TTX.

TTX standard (assay 98.3%) was provided by the Third Institute of Oceanography State Oceanic, Fujian, China. The 11-[^3^H]TTX (Figure 8, radiochemical purity 90.71%, specific radioactivity 9.86 Ci/mmol) was prepared according to the previously published methods [28] with slight modifications. In brief, lyophilized TTX was transformed to 11-oxoTTX by dicyclohexylcarbodiimide (DCC), using H_3_PO_4_ as the catalytic agent. Then, the 11-oxoTTX was deoxidized by tritiated sodium borohydride and the resultant 11-[^3^H]TTX was obtained with high purity by HPLC.

### 4.2. Radiolabeled Study Drug

The study drug was adjusted to a final specific radioactivity of 2842.875 µCi/mg (6,311,183 dpm/µg) by dilution with non-radiolabeled TTX standard. The dosing form concentration was 6 µg/g, with a radioactivity concentration of 36,519,188 dpm/g. Based on the available LD_50_ of TTX in rats of about 10 µg/kg, the chosen dose of 6 µg/(16 µCi/kg) (TTX + 11-[^3^H]TTX) was expected to be tolerated.

### 4.3. Analysis of the Radiochemical Purity of TTX

The radioactive constituents were separated by HPLC equipped with an automatic fraction collector. The HPLC was performed on a Shimadzu UFLC-20A, using a Waters Xbridge Amide HILIC (Milford, MA, USA) column (4.6 × 100 mm, 3.5 µm) equilibrated with 30% solvent A (0.1% formic acid aqueous solution) and 70% solvent B (acetonitrile, J&K Scientific, Beijing, China). Consequently, the eluent was collected into scintillation vials at 30-s intervals. The radiochemical purity of the sample in each vial was recorded and analyzed by LSC.

### 4.4. Animals and Experimental Design

Sprague-Dawley (SD) rats (8–10 weeks old at the time of the experiment, 240 ± 50 g in weight) were obtained from the Beijing Weitong Lihua Experimental Animal Technology Co., Ltd. (Laboratory animal production license No.: SCXK [Beijing] 2012-0001, Beijing, China).

### 4.5. Pharmacokinetic Study

For pharmacokinetic studies, a group of 6 rats, half male and half female, with jugular vein catheterization (JVC), were each administered an intramuscular (i.m.) injection with an average 6 µg/(16 µCi/kg) body weight of [^3^H]TTX. Serial blood samples (~0.3 mL) were collected in heparinized tubes via the jugular vein before and at time points of 0.033, 0.083, 0.167, 0.5, 1.0, 2.0, 4.0, 8.0, 24.0, and 48.0 h after administration. Plasma was separated and stored frozen at −20 °C until analysis.

### 4.6. Urine and Feces Excretion Study

For urine and feces excretion studies, 6 rats, 3 male and 3 female, were each administered an i.m. injection with an average 6 µg/(16 µCi/kg) body weight of [^3^H]TTX. Samples of urine, feces and cage wash solution were collected between 0–8 h, 8–24 h (feces only 0–24 h), 24–48 h, and 48–72 h after administration. All samples were stored frozen at −20 °C until analysis.

### 4.7. Bile Excretion Study

For bile excretion studies, a group of 6 rats, half male and half female, with biliary duct cannulation (BDC), were each administered an i.m. injection of 6 µg/(16 µCi/kg) body weight of [^3^H]TTX. Samples were collected between 0 and 4 h, 4 and 8 h, 8 and 24 h, 24 and 48 h, and 48 and 72 h after administration. All samples were stored frozen at −20 °C until analysis.

### 4.8. Tissue Distribution Study

For tissue distribution studies, a group of 24 rats (to minimize animal use, tissue samples at the 72 h time point came from the rats used in the urine and feces excretion studies), half male and half female, were each administered an i.m. injection of 6 µg/(16 µCi/kg) body weight of [^3^H]TTX. Samples of tissue (heart, liver, spleen, lung, kidney, whole brain, skin [neck], fat [abdomen], skeletal muscle [non-injection leg femur], gastric wall, intestinal wall, thymus, test is epididymis and uterus ovary) and carcass were collected at time points of 0.5, 4, 24, and 72 h after administration. All samples were stored frozen at −20 °C until analysis.

### 4.9. Control Group

For the control group (a male rat), samples of whole blood, plasma, tissue, and carcass were collected at the 24 h time point; samples of urine and feces were collected between 0 and 24 h. All samples were stored frozen at −20 °C until analysis.

### 4.10. General Methods

#### 4.10.1. Determination of Total Radioactivity

Total radioactivity in plasma, urine, bile, cage wash, whole blood, tissue and carcass was determined by liquid scintillation counter (LSC) using a Model Tri-Carb 3110TR (Perkin Elmer, Waltham, MC, USA). External standard methods were used for quenching correction, with counts at least 5 min per sample. Set scintillation counting automatically counts per minute (CPM) were converted to decays per minute (DPM). The value of the corresponding sample of the control group was measured as the background of the instrument, with calibration of samples in the experimental group. The background signal of the instrument was set to zero.

To determine the total radioactivity of plasma and urine samples, two samples were used: one (about 0.05 g) was added to scintillation liquid (5 mL) and determined directly by LSC, while the other was dried and purified water (0.1 mL) was added to reconstitute then scintillation liquid (5 mL) was added, followed by LSC. Bile and cage wash levels were determined directly by LSC. Feces, whole blood, tissue and carcass were homogenized in 50% isopropanol, weighed 2 samples (about 0.1 g), then KOH (6 M, 100 μL) was added, heated in a water bath, and then cooled. Scintillation liquid (5 mL) was added after glacial acetic acid and methanol were used to reconstitute, followed by LSC.

#### 4.10.2. Sample Preparation for Metabolite Identification and Profiling

Plasma samples that came from the pharmacokinetic study group were pooled by time points of 0.083, 0.5, 1.0, 2.0 and 4.0 h, mixing equal volumes of plasma from male and female rats at each time point. Each pooled plasma sample was extracted with 0.1% formic acid-methanol, after which the extracted solution was dried by nitrogen and the mixture of 0.1% HOOC–99.9%CH_3_OH:H_2_O (1:1, *v*/*v*) was added to reconstitute. Urine samples that came from the urine excretion study group were pooled between 0 and 8.0 h, mixing equal volumes of urine from male and female rats. Each pooled urine sample was precipitated with 0.3% formic acid–methanol, centrifuged, and then separated into solution.

Feces samples that came from the feces excretion study group were pooled between 0 and 24.0 h, mixing equal weights of feces from male and female rats. Each pooled feces sample was extracted with 0.1% formic acid–methanol, after which the extracted solution was dried by nitrogen and the mixture of 0.1% HOOC–99.9%CH_3_OH:H_2_O (1:1, *v*/*v*) was added to reconstitute. The radioactivity of the prepared samples was determined by LSC and metabolites were identified by LC-MS/MS.

#### 4.10.3. Pharmacokinetic Analysis

According to the radioactivity of plasma samples, the following main pharmacokinetic parameters were analyzed using the non-compartmental pharmacokinetics data analysis software WinNonlin (Version 6.3, Pharsight Corp., Los Angeles, CA, USA): maximum concentration (C_max_), time-to-maximum concentration (T_max_), area under the curve from 0 to the time (AUC_0–t_) and to the last measurable plasma concentration point (AUC_0–∞_), half-life (t_½_), and mean residence time (MRT).

#### 4.10.4. Metabolite Identification and Profiling

Prepared plasma, urine and feces samples were separated by HPLC equipped with an automatic fraction collector to collect eluent at 30-second intervals into scintillation vials, using LSC to analyze and convert by ARC^®^ Convert software (Los Angeles, CA, USA), to obtain reconstituted radioactive metabolite profiles. The spectrum of the radioactive metabolites of each sample was treated by integrals to obtain the peak area. The percentage of each metabolite and total radioactivity of each sample was calculated according to the peak area (%HPLC).

HPLC separation was performed on a Shimadzu UFLC-20A, using a Waters Amide HILIC column (4.6 × 100 mm, 3.5 µm) equilibrated with 20% solvent A (0.1% formic acid aqueous solution) and 80% solvent B (acetonitrile) at a flow rate of 1 mL/min. After 3 min, a linear gradient was run to 60% solvent A over 7 min and maintained for 3 min, followed by a linear gradient to 20% solvent A over 2 min and maintained for 9 min.

Analysis and identification of metabolites was conducted using a Thermo LTQ Orbitrap XL LC-MS/MS System (Thermo Scientific, Waltham, MA, USA) equipped with electrospray ionization (ESI). Detection was performed in positive ion mode under the following conditions: ion spray voltage at 3500 V, 350 °C, skimmer Offset at 10 units, tube lens offset at 80 units, sheath gas pressure at 20 units, auxiliary gas pressure at 10 units, sweep gas flow at 10 units, collision energy (CE) at 35 eV, and scan type at Q1 MS, MS/MS. Xcalibr software (Thermo Scientific, Waltham, MA, USA) was used for system control and data acquisition.

## 5. Conclusions

The present study investigated ADME of a single intramuscular dose of [^3^H]-radiolabeled TTX in adult Sprague-Dawley rats. The dose was rapidly absorbed and distributed mainly in the stomach, lungs, kidney and intestines. It was eliminated quickly and was unable to be detected in the plasma 24 h after administration. It also demonstrated that oxidation of TTX is the identified metabolite, except prototype drug (TTX). The results provide key reference information for the design and optimization of clinical trials of TTX.

## Figures and Tables

**Table 1:** Stability of 11-[^3^H]TTX.

Dose Solution Name	Time Points	Radiochemical Purity (%)
11-[^3^H]TTX	Pre-dose A (0 h)	90.17
	Post-dose B (4 h)	90.80
	Average	90.48

**Table 2:** Drying loss rate in plasma (%).

Time	Drying Loss Rate (%)
0	NO
2 min	13.71 ± 10.04
5 min	12.98 ± 4.60
10 min	7.45 ± 4.95
30 min	29.16 ± 3.48
1 h	38.65 ± 5.40
2 h	36.90 ± 5.54
4 h	64.85 ± 7.92
8 h	75.28 ± 5.37
24 h	100.00 ± 0.00
48 h	100.00 ± 0.00

Loss drying rate (%) = (Concentration before drying − Concentration after drying)/Concentration before drying × 100%. Values are presented as mean ± SD. SD, standard deviation (for three male and three female rats).

**Table 3:** Pharmacokinetic parameters of total radioactivity in dried plasma.

Parameter	Total Radioactivity in Dried Plasma		
	Male	Female	Average
T_max (h)_	0.11 ± 0.05	0.11 ± 0.04	0.11 ± 0.04
C_max_^a^ (ng Eq./mL)	5.08 ± 0.40	6.05 ± 1.78	5.56 ± 1.27
AUC_0–t_^a^ (h·ng Eq./mL)	5.50 ± 0.54	6.28 ± 0.17	5.89 ± 0.56
AUC_0–∞_^a^ (h·ng Eq./mL)	5.77 ± 0.55	6.83 ± 0.21	6.30 ± 0.69
MRT_0–t_ (h)	1.55 ± 0.09	1.68 ± 0.14	1.62 ± 0.12
t_½_ (h)	2.03 ± 0.14	2.59 ± 0.32	2.31 ± 0.38

^a^ In the calculation C_max_ and AUC, 1 g plasma according to 1 mL calculation. Values are presented as mean ± SD. SD, standard deviation (for three male and three female rats).

**Table 4:** Cumulative recovery rate of radioactivity (%) (0–72 h).

Samples	Over Time (h)	Recovery Rate (%)
Urine	0–8	46.69 ± 13.75
	0–24	50.01 ± 13.34
	0–48	51.09 ± 13.20
	0–72	51.16 ± 13.04
Feces	0–24	2.81 ± 1.59
	0–48	3.87 ± 1.88
	0–72	3.87 ± 1.88
Cage wash	0–24	9.55 ± 9.47
	0–48	9.82 ± 9.56
	0–72	10.01 ± 9.78
Carcass	72	4.31 ± 0.80

Values are presented as mean ± SD. SD, standard deviation (for three male and three female rats).

**Table 5:** Drying loss rates in urine (0–72 h).

Over Time Point (h)	Drying Loss Rate (%)
0–8	31.01 ± 8.05
8–24	41.83 ± 19.78
24–48	85.75 ± 34.92
48–72	100.00 ± 0.00

Loss drying rate (%) = (Concentration before drying − Concentration after drying)/Concentration before drying × 100%. Values are presented as mean ± SD. SD, standard deviation (for three male and three female rats).

**Table 6:** Cumulative recovery rates (%, SD-BDC rats; 0–72 h).

Samples	Over Time (h)	Recovery Rate (%)
Bile	0–4	0.29 ± 0.08
	0–8	0.42 ± 0.09
	0–24	0.43 ± 0.08
	0–48	0.43 ± 0.08
	0–72	0.43 ± 0.08
Urine	0–8	47.98 ± 11.10
	0–24	55.76 ± 13.39
	0–48	57.04 ± 13.36
	0–72	57.26 ± 13.19
Feces	0–24	0.47 ± 0.72
	0–48	0.94 ± 1.02
	0–72	0.94 ± 1.02
Cage wash	0–24	5.10 ± 2.23
	0–48	6.24 ± 2.65
	0–72	6.94 ± 2.82

Values are presented as mean ± SD. SD, standard deviation (for three male and three female rats).

**Table 7:** The contribution of TTX and metabolic No. 1 (M1) to the total [^3^H] radioactivity (100%) in the plasma of rats at different time points (%).

^3^H Resource	0.083 h		0.5 h		1 h		2 h		4 h	
	Female	Male	Female	Male	Female	Male	Female	Male	Female	Male
TTX	17.9	18.8	25.9	22.3	18.2	21.0	24.1	17.9	24.8	32.1
M1	1.80	4.10	4.94	5.75	5.51	3.35	3.35	1.80	4.81	4.26

Note: The percentages in Table 7 refer to the ratio of compound’s [^3^H] signal intensity to the total intensity of [^3^H] radioactivity LC radiochromatogram, measured by peak area normalization method.

**Table 8:** The contribution of TTX and metabolic No. 1 (M1) to the total [^3^H] radioactivity (100%) in the urine and feces of rats at different time points (%).

^3^H-Resources	Urine (0–8 h)		Feces (0–24 h)	
	Female	Male	Female	Male
TTX	26.35	26.11	28.19	12.38
M1	7.15	9.43	10.73	12.16

Note: The percentage in Table 8 refers to the ratio of compound’s [^3^H] signal intensity to the total intensity of [^3^H] radioactivity in the LC radiochromatogram, measured by peak area normalization method.
